# Supplementary material for: Smooth Interpolating Curves with Local Control and Monotone Alternating Curvature
Source: Comput Graph Forum. 2022 Oct 6;41(5):25–38. doi: 10.1111/cgf.14600 (PMC9827861; doi:10.1111/cgf.14600)
Supplement: Supplementary file 1 — Supplement Material [file CGF-41-25-s001.zip › Local-Smooth-Interpolating-MonoCurvature/extern/clothoids/submodules/Utils/ThirdParties/Eigen/eigen-3.3.9.zip!/eigen-3.3.9/doc/eigendoxy_header.html.in]

$projectname: $title
$title

$treeview
$search
$mathjax


Please, help us to better know about our user community by answering the following short survey: https://forms.gle/wpyrxWi18ox9Z5ae9

|  |  |  |  |
| --- | --- | --- | --- |
|  |
 $projectname  $projectnumber  $projectbrief |

 $projectbrief |


 $searchbox |
